# A multicomponent prehabilitation pathway to reduce the incidence of delirium in elderly patients in need of major abdominal surgery: study protocol for a before-and-after study

**DOI:** 10.1186/s12877-019-1101-7

**Published:** 2019-03-20

**Authors:** Ties L. Janssen, Christina A. Mosk, Chantal C. H. A. van Hoof-de Lepper, Daphne Wielders, Tom C. J. Seerden, Ewout W. Steyerberg, Adriaan J. van Gammeren, Dominique C. de Lange, René van Alphen, Martine van der Zee, René M. de Bruijn, Jolanda de Vries, Jan H. Wijsman, Gwan H. Ho, Paul D. Gobardhan, Lijckle van der Laan

**Affiliations:** 1 Department of Surgery, Amphia Hospital, P.O. Box 90518, 4800 RK Breda, The Netherlands; 2 Department of Gastroenterology, Amphia Hospital, Breda, The Netherlands; 3 Department of Public Health, Erasmus MC-University Medical center Rotterdam, Rotterdam, The Netherlands; 4 Department of Clinical Chemistry and Hematology, Amphia Hospital, Breda, The Netherlands; 5 Department of Geriatrics, Amphia Hospital, Breda, The Netherlands; 6 Department of Physical Therapy, Amphia Hospital, Breda, The Netherlands; 7 Department of Dieticians, Amphia Hospital, Breda, The Netherlands; 8 Medical Manager Surgery, Amphia Hospital, Breda, The Netherlands; 9 Department of Medical Psychology, Elisabeth-TweeSteden Hospital, Tilburg, The Netherlands; 10 Department of Medical and Clinical Psychology, Tilburg University, Tilburg, The Netherlands

**Keywords:** Prehabilitation, Multicomponent, Prevention, Delirium, Geriatric patient, Colorectal surgery, Abdominal aortic aneurysm, Quality of life

## Abstract

**Background:**

Due to the increase in elderly patients who undergo major abdominal surgery there is a subsequent increase in postoperative complications, prolonged hospital stays, health-care costs and mortality rates. Delirium is a frequent and severe complication in the ‘frail’ elderly patient. Different preoperative approaches have been suggested to decrease incidence of delirium by improving patients’ baseline health. Studies implementing these approaches are often heterogeneous, have a small sample and do not provide high-quality or successful strategies. The aim of this study is to prevent postoperative delirium and other complications by implementing a unique multicomponent and multidisciplinary prehabilitation program.

**Methods:**

This is a single-center controlled before-and-after study. Patients aged ≥70 years in need of surgery for colorectal cancer or an abdominal aortic aneurysm are considered eligible. Baseline characteristics (such as factors of frailty, physical condition and nutritional state) are collected prospectively. During 5 weeks prior to surgery, patients will follow a prehabilitation program to optimize overall health, which includes home-based exercises, dietary advice and intravenous iron infusion in case of anaemia. In case of frailty, a geriatrician will perform a comprehensive geriatric assessment and provide additional preoperative interventions when deemed necessary. The primary outcome is incidence of delirium. Secondary outcomes are length of hospital stay, complication rate, institutionalization, 30-day, 6- and 12-month mortality, mental health and quality of life. Results will be compared to a retrospective control group, meeting the same inclusion and exclusion criteria, operated on between January 2013 and October 2015. Inclusion of the prehabilitation cohort started in November 2015; data collection is ongoing.

**Discussion:**

This is the first study to investigate the effect of prehabilitation on postoperative delirium. The aim is to provide evidence, based on a large sample size, for a standardized multicomponent strategy to improve patients’ preoperative physical and nutritional status in order to prevent postoperative delirium and other complications. A multimodal intervention was implemented, combining physical, nutritional, mental and hematinic optimization. This research involves a large cohort, including patients most at risk for postoperative adverse outcomes.

**Trial registration:**

The protocol is retrospectively registered at the Netherlands National Trial Register (NTR) number: NTR5932. Date of registration: 05-04-2016.

## Background

The world’s population is aging, which subsequently leads to an increase in age-related diseases and conditions. Two major age-related diseases are colorectal carcinoma (CRC) and abdominal aortic aneurysm (AAA), which account for a large percentage of complications and prolonged length of hospital stay [1]. CRC is the third most frequent oncologic disease in the world in both men and women [2–4], with over 50% of patients being older than 70. People between 70 and 74 years old are most frequently diagnosed with CRC [3]. The cornerstone for treating CRC remains laparoscopic or open surgery [5]. Prevalence of AAA increases with age and ranges from 1.3% in women to between 4 and 7.7% in men over 65 years, with men having a six-fold greater risk [6]. AAA can be surgically treated via either open or endovascular aortic repair.

Up to 30% of patients undergoing major abdominal surgery and up to 35% of patients in CRC surgery develop postoperative complications [7, 8]. Old age increases the risk of complications and unplanned readmissions, which in turn lead to a longer hospital stays, higher mortality rates and a decrease in quality of life [8, 9].

Physical resilience decreases with age, while frailty increases [10]. Frailty is defined as a state of increased vulnerability which makes the ability to cope with the physical stress associated with surgery and other acute stressors compromised [11]. Incidence rates of frailty have been described of up to 43% in the population of CRC patients [12]. These frail patients have a four-fold greater risk of major postoperative complications [13], with longer hospital stay and higher 30-day readmission rates after both colorectal surgery and abdominal aortic repair [14, 15]. Delirium is a frequent postoperative complication in the frail elderly population, with incidence rates described of 25% after major elective surgery and up to 50% after high-risk procedures [16, 17].

Despite advances such as minimally invasive surgery, protocols to prevent surgical site infection and fast-track protocols, which have decreased the impact of surgically induced trauma and the number and severity of postoperative complications, incidence rates of delirium remain high [18–22]. Additionally, many short- and long-term complications are still observed and occur in elderly patients especially, with even worse outcomes in the frail [23–25]. Several preoperative programs have been suggested to tackle factors of frailty and further reduce postoperative complications such as delirium in the elderly population. Some programs are specifically developed to prevent delirium, since delirium is independently associated with serious adverse outcomes such as functional decline, cognitive decline, increased length of hospital stay and ICU stay, institutionalization, increase of health-care costs and increased mortality rates [24, 26].

Primary prevention is the most effective strategy for minimizing occurrence of delirium as well as delirium-associated complications [27], with favorable outcomes in multicomponent (non-pharmacological) interventions as concluded by two systematic reviews and meta-analyses [28, 29]. Although of questionable quality, different pharmacological and non-pharmacological preoperative preventive approaches have been trying to further reduce the incidence of postoperative delirium during admission. Pharmacologic interventions proved unsuccessful and the quality of current evidence for improvement with non-pharmacological approaches was labeled moderate [28].

Over the past few years, different studies have suggested ‘prehabilitation’ programs to reduce postoperative complications. Prehabilitation is a preparatory intervention, prior to admission, aiming at optimizing patients’ physiologic reserves in anticipation of a forthcoming physiological stressor and minimizing peri- and postoperative adverse events [7]. Uni-, bi- and trimodal approaches have been investigated to tackle factors of frailty and to reduce postoperative complications in both CRC and AAA patients [7, 28–40]. The main focus was to improve muscle strength, cardiopulmonary condition, undernourishment or psychological problems, often with functional capacity as primary outcome. All studies performed were heterogeneous in composition and factors they tried to influence [37], which makes it impossible to pool results and provide high-quality evidence. Therefore, two recent systematic reviews concluded that there currently is no clear evidence showing that improved preoperative fitness will translate into a decrease in postoperative complications [7, 39].

Previous prehabilitation trials often involved a small number of patients undergoing the prehabilitation program, not exceeding 75 patients. These trials did not focus on elderly patients specifically, with a mean age often below 70. Few studies used health related quality of life as primary or secondary outcome and none of these studies focused specifically on preventing postoperative delirium [37].

Up to 75% of patients with CRC suffer from iron deficiency anaemia and even mild anaemia can have impaired functional capacity or postoperative delirium as a consequence [24, 40, 41]; yet hematinic optimization is often not included in a multicomponent prehabilitation pathway [42]. Patients receiving perioperative red blood cell transfusions because of this anaemia have an increased risk of adverse clinical outcomes, including increased delirium and mortality rates [43, 44]. It is therefore important to optimize hemoglobin levels before surgery and prevent the need for this transfusion.

The aim of this study is to reduce the incidence of delirium by implementing a unique combined pharmacological and non-pharmacological multidisciplinary program in order to optimize overall fitness in the elderly patients before CRC resection or AAA repair. By implementing this preoperative program, combined with the above-mentioned SSI and ERAS protocols, the primary goal is to reduce the incidence of postoperative delirium. Secondary goals are to reduce other postoperative complications, shorten hospital stay, prevent unplanned ICU admission, reduce mortality rates and improve prehabilitated patients’ quality of life postoperatively. If reduction of delirium incidence proves successful, a health-economic analysis will be performed to assess cost-effectiveness. If subsequent results prove (cost-) effective, nationwide implementation is the objective.

## Methods

### Design and setting of the study

This protocol describes a single-center controlled before and after study with an intention–to–treat design. A unique multidisciplinary care pathway is designed, starting at the 70PLUS outpatient clinic of the department of surgery of the Amphia hospital Breda, a tertiary teaching hospital in the Netherlands. Table 1 provides an overview of the complete study period, from inclusion to 12 months follow-up.

**Table 1:** Complete overview of the study period

Time point	Study period								
	Eligibility assessment		Trial enrollment	70PLUS outpatient clinic	Admission	Discharge	Discharge + 2 weeks	6-months follow-up	12-months follow-up
	Pathology result/ CTA	Multidisciplinary meeting	T0 – Informed consent obtained	T1	T2	T3	T3.5	T4	T5
Assessments									
Laboratory testing				X	X	X			
Nurse practitioner or investigator									
Baseline patient characteristics				X					
Factors of frailty				X					
MMSE				X		X		X	X
CCI and ASA				X					
P-POSSUM				X					
ISAR-HP				X				X	X
PARKER				X				X	X
SNAQ				X					
KATZ-ADL				X				X	X
Caregiver burden				X			X	X	X
CESD-16				X			X	X	X
WHOQOL-BREF				X			X	X	X
Physiotherapist									
10MWT				X					
TCST				X					
TUG				X				X	X
MIP				X					
Handforce				X					
Dietician									
MNA-SF	Indications for referral to dietician: Unintentional weight loss Loss of appetite BMI < 22 Undernourishment			X					
BMI				X				X	X
SNAQ				X					
Geriatrician									
Comprehensive geriatric assessment	Indications for referral to geriatrician: Delirium in history MMSE ≤24 TUG ≥12.6 s Polypharmacy			X					
Interventions									
Laboratory testing / Intravenous iron suppletion									
All patients	Single dose of 1000 mg Ferric carboxymaltose (Ferinject®) at day care when indicated: Hb level males < 8,1 mmol/L Hb level females < 7,4 mmol/L			X					
Physiotherapist									
All patients	30 min of daily walking or cycling 5 exercises to improve leg muscle strength 2 × 15 minutes respiratory muscle exercise Transfer training when indicated (getting out of bed)			X					
Dietician									
Malnourished patients / MNA-SF < 12	Dietary advice on required protein and calorie intake. Proteins: 1.2 g/kg bodyweight (BMI < 30) Calories: WHO formula for basal need + 30%. Supplements are provided when required protein and calorie intake is not met after dietary advice.			X					
Geriatrician									
Frail patients	Non-pharmacological interventions to reduce risk of delirium. Pharmacological interventions (prophylaxis).			X					
Time from pathology result to multidisciplinary meeting: < 1 week. Time from multidisciplinary meeting to T0: < 1 week. Time from T0 to T1: < 1 week. Time from T1 to admission: 10 days to 5 weeks									

*MMSE* Mini-Mental State Examination, *CCI* Charlson Comorbidity Index, *ASA* American Society of Anaesthesiology, *P-POSSUM* Portsmouth Physiological and Operative Severity Score for the Enumeration of Mortality and Morbidity, *ISAR-HP* Identification of Seniors At Risk – Hospitalized Patients, *CES-D16* Centre for Epidemiological Studies – Depression 16 questions, *WHOQOL-BREF* World Health Organisation Quality of Life – BREF, *10MWT* 10-m Walk Test, *TCST* Timed Chair Stand Test, *TUG* Timed-up and Go Test, *MIP* Maximum Inspiratory Pressure, *MNA-SF* Mini Nutritional Assessment – Short Form, *BMI* Body Mass Index, *SNAQ* Short Nutritional Assessment Questionnaire

### Patient characteristics and recruitment

All patients aged 70 and older who are scheduled to undergo elective abdominal surgery in case of CRC or AAA, at the Amphia hospital are assessed for eligibility. They will undergo robot-assisted laparoscopic resection, laparoscopic resection or open removal of the colorectal tumor, or EVAR or open aortic repair of their AAA.

Patients are considered ineligible if acute hospitalization or acute surgery is needed (necessity established by the gastroenterologist, gastrointestinal surgeon or vascular surgeon), if they had surgery 6 months prior or if surgery is planned within 2 weeks of the multidisciplinary meeting.

Eligibility for participation is established at the multidisciplinary meetings for colorectal cancer and for vascular surgery. After this establishment, patients are invited to participate by the gastroenterologist or the vascular surgeon. If preoperative chemotherapy, radiotherapy or chemoradiation is indicated, patients are included in the study when the indication for surgery is made, prior to these neoadjuvant therapies. Patients receive oral and written information about the study. If patients agree to participate, they are invited by the primary investigator to visit the 70PLUS outpatient clinic. Written informed consent will be obtained from all study participants during this visit. Due to the design of the study and the electronic patient file, it is not possible to blind participants, investigators, care providers, or outcome assessors in this study.

### The 70PLUS outpatient clinic

All patients will visit a trained nurse practitioner and a physical therapist at the 70PLUS outpatient clinic. A dietician and a geriatrician will be consulted in case of undernourishment or frailty respectively. Each visit to the healthcare providers takes approximately 1 hour. The visit to a geriatrician is planned on a separate day because of the major burden of a four-hour visit.

In the optimal situation, patients have 5 weeks prior to surgery to optimize their overall fitness, starting from the moment they visit the 70PLUS outpatient clinic. Patients in need of neoadjuvant treatment will have a longer optimization period, however this advantage will likely be nullified by the burden of these treatments. Patients are invited to visit the outpatient clinic when indication for surgery is made. Time of surgery is based on the surgical program and physical complaints of the patient at that moment.

The nurse practitioner screens for frailty and determines the need for consulting the other physicians. Indications for consulting these physicians are described in Table 1. The following baseline patient characteristics are assessed: age, gender, surgical and general medical history, comorbidities, use of medication, intoxications, social economic status and schooling, body mass index, home situation (need for home care, institutionalization and social environment), functional dependency (Parker score and Identification of Seniors At Risk – Hospitalized Patients (ISAR-HP) score), psychological history and burden of comorbidity (the American Society of Anesthesiologist (ASA) score, Portsmouth Physiological and Operative Severity Score for the enUmeration of Mortality and Morbidity (P-POSSUM score) and the Charlson comorbidity index (CCI)) [45–48].

Frailty is screened for by collecting information on visual or hearing impairment, sleep rhythm, feeding impairment, dehydration and fall risk. Additional information on frailty is acquired by performing the following questionnaires: the delirium screening checklist, the Groningen frailty score, the KATZ-Activities of Daily Living score (for assessment of functional dependency and mobility) and the Short Nutritional Assessment Questionnaire (SNAQ) (for assessment of nutritional status) [49–51].

A district nurse assesses the need for additional homecare postoperatively for each patient. This assessment will be applied for by the nurse practitioner for all patients who commit to this pathway, in order to shorten the length of hospital stay.

### Physiotherapist visit and assessment of physical condition

At initial assessment, the physiotherapists measure the patients’ cardiopulmonary condition, overall strength and frailty.

Gait, stability and speed are assessed by the Timed Up and Go (TUG) test and the 10-m walking test. These tests are validated and widely used in elderly patients. A cutoff of 12.6 s is used to define frailty. If considered frail, a geriatrician is consulted. TUG has been found to be superior to ASA-score in identifying oncogeriatric patients who might benefit from a prehabilitation program [52–54]. The timed-chair-stand test is used to assess lower extremity strength, specifically vertical movement and hip muscle strength [55].

Strength of diaphragm and inspiratory muscles is assessed by measuring the maximum inspiratory pressure (MIP) during 1.5 s, using MicroRPM™. Training of inspiratory muscles successfully reduces length of hospital stay and postoperative pulmonary complications [56].

Strength of both hands is tested using Hydraulic Hand dynamometer, JAMAR™. Poor handgrip strength is associated with a decline in activities of daily living and cognition in the elderly [57].

In order to lower the burden for patients, lower the health-care costs and increase compliance, the prehabilitation program has a large home-based component. All patients receive personalized exercises to preserve or improve their overall fitness and strength at home, unsupervised. These exercises have to be performed daily. The aim of these exercises is to increase respiratory muscle strength using the Threshold inspiratory muscle trainer®, and to improve overall fitness by endurance training, which consists of daily walking or cycling for 30 consecutive minutes. Patients receive specific transfer exercises and specific exercises to increase muscle strength in both legs and arms. All exercises are individualized to each patient’s capabilities, as not every patient has the same baseline motivation or fitness.

Patients are asked to keep a diary with a record of their daily activities to assess compliance with the prehabilitation program. A cut-off value of 75% or more was considered compliant with the training program.

### Dietician consultation and nutritional assessment

Nutrition is quantified using the body mass index (BMI), the Mini Nutritional Assessment score short form (MNA-SF) and the SNAQ-score [51, 58]. Laboratory research assesses blood levels of folic acid, vitamins B and D, lipid-spectrum and pre-albumin. Indications for consulting the dietician are described in Table 1. Based on the hospital’s protocol for patients who will undergo major abdominal surgery, the dietician provides supplemental protein drinks and dietary advice if needed.

### Cognition and mental health assessment

The patient is labeled frail, or at increased risk of developing delirium, if any form of cognitive impairment has previously been diagnosed. Cognition is examined using the Mini-Mental State Examination (MMSE), a standardized questionnaire designed specifically for this purpose [59]. The MMSE has a sensitivity of up to 97% and specificity of up to 70%, when adjusted for educational level [60]. A score below 24 or 26 points, depending on education level, is also considered an indication of frailty. In these increased-risk cases (see Table 1), the geriatrician will perform a comprehensive geriatric assessment (CGA) [61, 62].

The CGA is an effective method to identify patients with increased risk for postoperative complications [61–64]. In the CGA, a geriatrician will assess if additional preventive intervention is necessary (e.g. prescribing prophylactic haloperidol, critically reviewing mediation, and providing advice on non-pharmacologic prevention of infection, falls, pain, anxiety and dehydration). Prophylactic haloperidol to be given during admission is prescribed to cognitively impaired patients and patients with delirium in medical history. The CGA is effective in improving mortality rates after 36 months of follow-up, improving functional independence and physical function and in decreasing rates of institutionalization [65].

Depression is screened for by using the CESD-16 questionnaire, which is a shortened version of the CESD-20 [66, 67]. Caregiver burden will be assessed by using the caregiver strain index questionnaire [68].

Quality of life is assessed using the WHOQOL-BREF. This is a shorter version of the WHOQOL-100, a questionnaire introduced by the World Health Organization. This questionnaire does not assess the physical capabilities of a patient, but assesses the patient’s opinion of having or not having these capabilities. This way, it provides a better display of a patient’s quality of life compared to questionnaires such as the SL-36, which shows a patient’s functional capacity [69, 70].

### Biochemistry

Blood is collected from patients at or just before their first visit to the outpatient clinic. The following concentrations are determined via laboratory research: hemoglobin, hematocrit, leukocytes, thrombocytes, MCV, erythrocytes, INR, CRP, sodium, potassium, chloride, urea, creatinine, GFR, pre-albumin, ASAT, ALAT, cholesterol, HDL cholesterol, LDL cholesterol, triglycerides, iron, transferrin, ferritin, folic acid, vitamin B12, 25-OH-Vitamin D and CEA.

Anemic patients (hemoglobin level of < 7.4 mmol/L (< 120 g/L) for women and < 8.1 mmol/L for men (< 130 g/L)) receive a single dose of 1000 mg Ferric carboxymaltose (Ferinject®) preoperatively to increase hemoglobin levels [71–73]. This is the fastest and safest way of correcting preoperative hemoglobin levels in patients suffering from iron deficiency anaemia [74].

Blood collection and laboratory research will be repeated at admission (preoperative) and at the day of discharge (postoperative).

Table 1 provides a complete overview of cooperating physicians, indications for consulting these physicians, and actions and questionnaires performed by these physicians.

### Admission

For both the control group and the intervention group, standard preventive measures for delirium will be taken during admission according to the HELP guidelines and postoperative patient care will be provided according to ERAS protocols [75].

### Follow up

Patients will be asked to visit the outpatient clinic at six and 12 months after discharge. These visits will last no longer than 20 min. The WHOQoL-BREF, CESD-16, MMSE, TUG, BMI, KATZ-ADL, PARKER and ISAR-HP are scored. Emergency department visits, readmission since initial discharge and deaths during follow-up will be registered. When trial participation is discontinued postoperatively, follow-up data on complications, readmissions, institutionalization and mortality will be acquired through retrospective chart review.

### Study outcomes

The primary study outcome is the incidence of delirium. Delirium will be screened for with the Delirium Observational Screening Score (DOSS), using the shortened version which consists of 13 items [76]. The DOSS is scored three times every day. A delirium is likely if the patient has a DOSS of ≥3. If delirium is suspected, a geriatrician will confirm the diagnosis using the DSM-V criteria and the confusion assessment method (CAM) [77, 78].

The secondary outcomes are postoperative length of hospital stay, ICU admission, readmissions, institutionalization, mortality within 1 year after surgery, and quality of life. Number and severity of postoperative complications during hospital stay and follow-up will be assessed and scored according to the Clavien-Dindo classification [79, 80].

All other factors of frailty, that are mentioned in sections 2.3 to 2.7, will be evaluated and analyzed to confirm association with the incidence of delirium.

### Statistical analysis

The sample size was calculated based on data from a previous study [24]. Based on the analysis of 232 patients with AAA or CRC, this study found a delirium incidence of 15%. A 50–50 trial needs 550 patients, or 275 patients per study arm, to reduce the incidence of delirium to 7.5%. These calculations are based on a power of 80% with a 5% two–sided significance level.

Starting in November 2015, the aim is to include 275 patients in the prospective study, which is feasible during approximately 4.0 years of accrual, including follow-up. These patients will be compared to a group of patients treated between January 2013 and October 2015 at the Amphia Hospital Breda, The Netherlands. This group is formed by applying the same inclusion and exclusion criteria as are used to include patients prospectively, they were given the same perioperative care as given to the prehabilitation group, but they did not partake in any prehabilitation program. All baseline characteristics and postoperative outcomes mentioned in previous sections for the control group will be acquired through retrospective chart review, however due to the design of this research it is likely that not all these characteristics have been documented. Data acquired during the six- and 12 months follow-up visits have not been collected for the control group and will only be used for analyses on the prehabilitation group. For example, blood levels cannot be determined through retrospective chart review, however functional dependency can be acquired. The ratio of open versus minimally invasive surgery is expected to be the same in both groups, since conditions and indications for minimally invasive procedures did not change over study time.

Descriptive statistics will be used for presenting baseline characteristics. Differences in these characteristics between the control group and the prehabilitation group will be tested for statistical significance by using Student t-test or Mann-Whitney U test for continuous variables and Pearson chi-squared test or Fisher’s Exact test for categorical variables, depending on normality. A subgroup analysis will be performed per diagnosis to test for differences between the groups in the primary and secondary outcomes.

Primary analysis for incidence of delirium will be done by creating a logistic regression model with adjustment for age, history of delirium, ASA ≥3 and diagnosis (AAA or CRC), important prognostic covariates found in previous research [24]. Secondary outcomes will likewise be adjusted for these covariates, however history of delirium will be replaced with type of surgery (open or minimally invasive) [81]. Mixed linear modeling will be applied for measurements over time during follow up. Missing retrospective data will not be multiply imputed.

All data will be gathered in the Amphia Hospital Breda, the Netherlands, using the electronic patient file ‘Hyperspace Version IU4 (Epic, Inc., Verona, WI)’.

Statistical analysis will be performed with IBM SPSS statistics software (SPSS Inc., Chicago, Illinois, USA). A two-sided *p*-value < 0.05 will be considered statistically significant. Data analysis will be done according to the intention-to-treat concept. Data collection is still ongoing.

This article was has been reported by making use of the SPIRIT guidelines [82].

### Patient and public involvement

Patients were not involved in the design of this study, selection of outcome measures, development of research question, and in the recruitment to and conduct of this study. The burden of the intervention will be assessed during follow-up. A brief summary of the results will be made available in Dutch or English to all patients on request.

## Discussion

This new and unique program is a multicomponent and multidisciplinary approach in order to optimize elderly patients in need of major abdominal surgery. The objective of this prehabilitation program is to decrease the incidence of delirium by tackling factors of frailty and by optimizing overall fitness, nutritional status, mental status and anaemia at the same time.

Dutch (SONCOS) guidelines for colorectal cancer suggest an *optimal* time of 6 weeks from diagnostic pathology results to surgery [83]. The time between the pathology result and the multidisciplinary meeting (MDM) and between the MDM and the 70PLUS outpatient clinic visit is no more than a week, leaving an ideal optimization period of approximately four to 5 weeks. Patients with obstruction or pain will be operated on as soon as possible, thus are not able to finish the entire prehabilitation period. There are no such guidelines for AAA surgery, meaning that this patient group should be able to complete the full 5 weeks of prehabilitation.

The combination of AAA and CRC was chosen for this study, even though they are different diseases with their own etiology. Both conditions are a heavy burden on a patient’s fitness and are major diseases for which abdominal surgery is required. This combination is justifiable because both diseases have similar surgical risk factors and factors of frailty that increase the risk for a delirium.

Patients are asked to perform unsupervised physical exercises at home, which might reduce compliance because there is no extrinsic motivator. By giving patients tailor-made exercises and by reducing the number of extra hospital visits, better compliance is to be expected [36].

The awareness for delirium in elderly surgical patients has increased in postoperative care, for both doctors and nurses, which might cause a relative increase in delirium cases over time. It is expected that the prehabilitation program will cancel out this effect.

### Strengths and limitations

Compared to other prehabilitation studies, this study aims to include a large prospective and retrospective patient cohort and focusses solely on patients that are > 70 years. A multimodal approach was implemented, combining both pharmacological and non-pharmacological interventions, involving physical fitness, nutritional status and hemoglobin levels. Previous studies often implemented a single intervention, within a small sample and mean age below 70 [30, 31, 34–37]. With increasing age, physical resilience starts to decrease, which in turn increases the need for prehabilitation, emphasizing the importance of prehabilitation in the elderly.

Due to logistic reasons, not all patients can be visited at admission. Timing of admission makes it impossible to verify improvements in overall fitness, pulmonary muscle strength or the effectiveness of the physical interventions in all patients. In the end, the focus of this research is to prevent delirium and other postoperative adverse events, making quantifying the progress made by the program irrelevant. Previous studies have proven similar programs to be able to make a significant improvement in functional capacity [37].

The design of this study makes the risk of bias fairly high. Due to the before-and-after setting, randomization and blinding of patients and caregivers is impossible. Bias may also occur because surgical procedures have changed over time, although no significant differences are expected due to the consecutive timing of both cohorts.

This study has a single-center design, which may limit generalizability of the study. However, the way this intervention is set-up makes it possible to implement it in other centers as well.

## Abbreviations

AAA: Abdominal aortic aneurysm
ASA: American Society of Anesthesiologist
BMI: Body mass index
CAM: Confusion assessment method
CCI: Charlson comorbidity index
CGA: Comprehensive geriatric assessment
CRC: Colorectal carcinoma
DOSS: Delirium observational screening score
ERAS: Enhanced recovery after surgery
EVAR: Endovascular aortic repair
HELP: Hospital Elder Life Program
ICU: Intensive care unit
ISAR-HP: Identification of Seniors At Risk - Hospitalized Patients
MDM: Multidisciplinary meeting
MIP: Maximum inspiratory pressure
MMSE: Mini-mental state examination
MNA-SF: Mini nutritional assessment score short form
P-POSSUM: Portsmouth Physiological and Operative Severity Score for the enUmeration of Mortality and Morbidity
SNAQ: Short nutritional assessment questionnaire
SSI: Surgical site infection
TUG: Timed up and go
WHOQOL: World health organization quality of life
